# High repetition rate green-pumped supercontinuum generation in calcium fluoride

**DOI:** 10.1038/s41598-021-94411-1

**Published:** 2021-07-22

**Authors:** Vaida Marčiulionytė, Vytautas Jukna, Gintaras Tamošauskas, Audrius Dubietis

**Affiliations:** Laser Research Center, Vilnius University, Saulėtekio Avenue 10, LT 10223 Vilnius, Lithuania

**Keywords:** Optics and photonics, Optical materials and structures

## Abstract

We compare supercontinuum generation in $\hbox {CaF}_2$ crystal under tight and loose focusing of 150 fs, 515 nm second harmonic pulses from an amplified Yb:KGW laser at a repetition rate of 10 kHz. It is demonstrated that supercontinuum generation geometry applying loose focusing ($\hbox {NA}=0.004$) of the pump beam into a long (25 mm) $\hbox {CaF}_2$ sample is advantageous in terms of supercontinuum spectral extent and durability of damage-free operation of the nonlinear material as compared to a commonly used supercontinuum generation setup which employs tight focusing ($\hbox {NA}=0.012$) into a short (5 mm) sample and to setup which uses tight focusing into a long (25 mm) sample. More specifically, loose focusing into a long sample showed remarkably longer (20 min) damage-free operation of the nonlinear material, which was not translated with respect of the pump beam, while in tight focusing condition the sample is damaged just within 2 min of operation, leading to a complete extinction of the supercontinuum spectrum. The evolution of optical degradation of the nonlinear material in time and its impact to supercontinuum spectrum is studied in terms of filament-induced luminescence due to self-trapped exciton emission and light scattering at the pump wavelength indicating the onset of optical damage. Our findings are supported by the numerical simulations which compare relevant parameters related to filament propagation in tight and loose focusing conditions.

## Introduction

Supercontinnum (SC) generation in the ultraviolet (UV) spectral range is a formidable task due to lack of suitable nonlinear solid-state materials^1–3^. The largest spectral broadenings and the largest spectral blue-shifts in the UV, in particular, are obtained when SC is generated in dielectric materials featuring large energy bandgap, i.e. materials having wide transparency in the UV and small chromatic dispersion^4,5^. These criteria are fulfilled in alkali metal fluorides: $\hbox {CaF}_2$, $\hbox {BaF}_2$, $\hbox {MgF}_2$, LiF and LiSAF, which indeed produce SC spectra with the largest spectral blue-shifts extending into deep UV, and show good performance with femtosecond near and mid infrared laser pulses^6–21^. Although alkali metal fluorides formally exhibit high optical damage thresholds as exposed to a single femtosecond laser pulse^22^, they all suffer from heat accumulation and color center formation that lead to rapid optical degradation due to defect accumulation and eventually, to optical damage under irradiation with repetitive pulses. The same applies to halide crystals, where formation of color centers was shown to strongly affect SC generation^23^. Therefore, to reduce the impact of color centers and achieve stable performance, these nonlinear materials have to be continuously translated or rotated with respect to the incident laser beam even at fairly low (e.g., 1 kHz) repetition rates.

Out of these, $\hbox {CaF}_2$ was demonstrated as the most reliable nonlinear material which produces broad and stable SC spectrum in practical setups employing near-infrared pump pulses (typically delivered by Ti:sapphire lasers). Its efficient performance was justified by practical applications in ultrafast time-resolved spectroscopy^24–26^ and production of femtosecond UV pulses via noncollinear optical parametric amplification^6,27,28^.

However, open questions remain for what concerns optimal experimental settings (i.e. focusing geometry and choice of pump wavelength) to achieve desired spectral extent of the SC, its long-term stability and the durability of the nonlinear material itself, that is, the performance time of the crystal without the onset of optical degradation. First of all, loose focusing proved to be beneficial in several relevant aspects: increased red-shifts of the SC spectra^29,30^ and improved pulse-to-pulse stability of spectral components^31^. Secondly, it is worth noticing that pump pulses with a shorter central wavelength (e.g., harmonics of solid-state lasers) produce SC spectra with relatively smaller blue-shifts, but shorter absolute cut-off wavelengths, see e.g.^19,20,32^. Thirdly, it was demonstrated recently that durability of the nonlinear material increases markedly when the pump pulses with a shorter wavelength are used^20^. This observation was attributed in part to quadratic wavelength dependence of the critical power for self-focusing, and in part due to the reduced contribution of impact ionization, whose rate decreases with decreasing the pump wavelength.

Considering the above, in this paper we study SC generation in $\hbox {CaF}_2$ with 150 fs, 515 nm pulses at a repetition rate of 10 kHz and compare the performance characteristics using tight ($\hbox {NA}=0.012$) and loose ($\hbox {NA}=0.004$) focusing geometries into thin (5 mm) and thick (25 mm) samples of the nonlinear material, respectively.

## Methods

### Experimental

**Figure 1:**
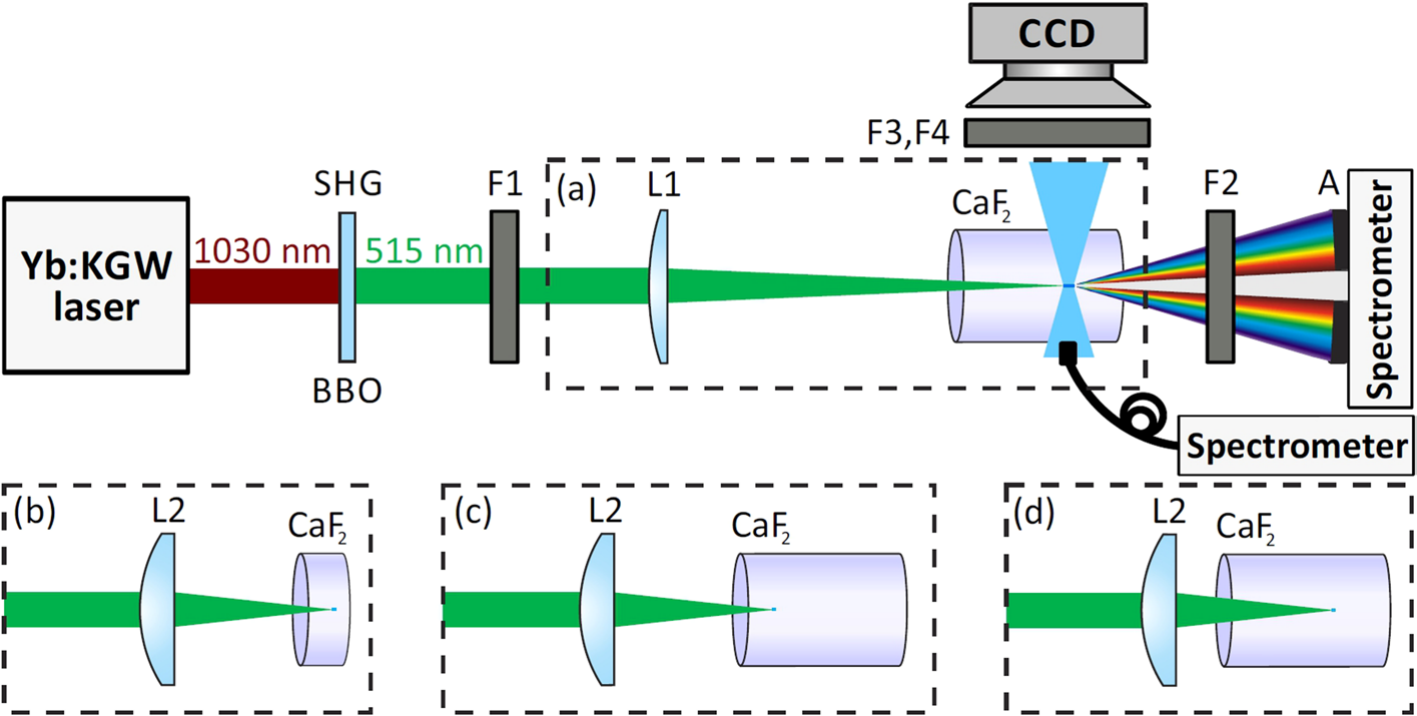
Schematic of the experimental setup. SHG: BBO crystal for the second harmonic generation, F1: filter for blocking the fundamental harmonic, L1 and L2: focusing lens, $\hbox {CaF}_2$ crystal samples of 25 mm and 5 mm thickness, A: iris aperture, F2: combined attenuator, F3, F4: filters for transmitting luminescence and scattering signals, respectively. Panels (**a**–**d**) illustrate focusing configurations of the pump beam. See text for details.

The experiment was performed with an amplified Yb:KGW laser (Pharos, Light Conversion) which operated at a 10 kHz repetition rate. The experimental setup is depicted in Fig. 1. The laser pulses with a duration of 180 fs and a central wavelength of 1030 nm were frequency doubled in 1-mm-thick beta barium borate (BBO) crystal cut for type I phase matching to produce 150 fs pulses at the second harmonic (515 nm). Filter F1 was used to cut the residual fundamental harmonic. The SC generation was investigated under loose ($\hbox {NA} = 0.004$) and tight ($\hbox {NA} = 0.012$) focusing geometries, which were realized by focusing the second harmonic beam of 3.5 mm diameter (at the $1/\textit{e}^{2}$ intensity level) by either L1 ($\textit{f} = +400$ mm) or L2 ($\textit{f} = +150$ mm) lenses, respectively. In the experiment we used $\hbox {CaF}_{{2}}$ samples of 25 mm and 5 mm thickness (provided by Eksma Optics); the samples were not translated during the measurement. 5 mm sample was used in tight focusing conditions ($\hbox {NA}=0.012$) and 25 mm sample was used in loose focusing conditions ($\hbox {NA}=0.004$). In both cases the pump pulse energies and sample positions with respect to the focusing lens were chosen to produce a single filament close to the exit face of the sample as illustrated in Fig. 1a,b, and to achieve saturation of spectral broadening and stable SC spectrum at the output. In order to make a comparison more convincible, we also performed a complimentary experiment using tight focusing ($\hbox {NA}=0.012$) into $\hbox {CaF}_{{2}}$ sample of 25 mm thickness. In that case we investigated two distinct focusing configurations, where the light filament was induced either close to the input or exit faces of long sample, as shown in Fig. 1c,d, respectively. An iris aperture A was used to block the conical emission and transmit only the axial portion of the SC radiation, which was coupled into a slit of the spectrometer (AvaSpec-3648, Avantes), that provides a detection range from 200 to 1100 nm. In order to increase the dynamic range of spectral measurements and prevent the saturation of the spectrometer detector, the most intense part of the SC spectrum (i.e. around the second harmonic wavelength) was attenuated by using an appropriate combination of dielectric mirrors and color filters, altogether labelled as F2. Thereafter the spectral data was corrected for the individual mirror and filter transmission functions and the sensitivity function of the spectrometer detector. A complimentary fiber spectrometer (QE65000, Ocean Optics) was used to measure spectrum of the filament-induced luminescence.

Along with spectral measurements, we recorded the time evolutions of filament-induced luminescence trace in the UV and light scattering at the pump wavelength inside the crystal, which were captured from polished sides of the samples, as illustrated in Fig 1. These measurements were performed by imaging the area around the nonlinear focus onto the CCD camera (Grasshopper2, Point Grey) with a pixel size of $4.4\,\mu \hbox {m}$ and using appropriate sets of color filters, labelled F3 (transmitting in the UV) and F4 (transmitting in the visible, around the pump wavelength), respectively.

#### Numerical

The numerical simulations were carried out using a model which solves a unidirectional nonparaxial propagation equation for the pulse envelope ${\hat{E}}(\omega ,k_{\perp },z)$, assuming revolution symmetry^33^:

1 $$\begin{aligned} \frac{\partial {\hat{E}}}{\partial z}= i\left( \sqrt{k^2(\omega )-k_{\perp }^2} -k_0-k_0'\omega \right) {\hat{E}}+ i\frac{\omega }{2n(\omega )c}\varepsilon _0^{-1}\left( {{\hat{P}}}+i\frac{{\hat{J}}}{\omega }\right) , \end{aligned}$$

where *c* is the speed of light in a vacuum, $\varepsilon _0$ is the vacuum permittivity, $k_{\perp }$ is the transverse wave vector, $k(\omega )$ is the dispersion relation of the medium, $n(\omega )$ is the refractive index of the medium calculated from a Sellmeier equation^34^. In the present case $n_0=1.436$ was used to evaluate $k_0\equiv k(\omega _0)$ and $k'_0\equiv dk/d\omega |_{\omega _0}$ at the central laser frequency $\omega _0$. The nonlinear polarization *P*(*t*, *r*, *z*) and current source *J*(*t*, *r*, *z*) terms were computed in the space-time domain:

2 $$\begin{aligned} \varepsilon _0^{-1}P= & {} 2n_0n_2|E|^2E, \end{aligned}$$

3 $$\begin{aligned} \varepsilon _0^{-1}J= & {} n_0c\left[ \sigma (1+i\omega _0\tau _c)\rho + \frac{WU_g}{|E|^2}\left( 1-\frac{\rho }{\rho _{nt}}\right) \right] E, \end{aligned}$$

where $n_2=1.9\times 10^{-16}\,\hbox {cm}^2/\hbox {W}$^35^ is the nonlinear refractive index, $\sigma =2.7\times 10^{-20}\,\hbox {m}^2$ is the cross section for inverse Bremsstrahlung, $\tau _c=2$ fs is the effective electron collision time, $\rho _{nt}=2.1\times 10^{22}\,\hbox {cm}^{-3}$ is the density of neutral molecules, and $\rho$ is the density of free electrons in the conduction band.

The intensity dependent photoionization rate *W* was calculated from Keldysh’s theory with electron-hole mass ratio $m^*=1$ and assuming band gap of $\hbox {CaF}_2\,U_g=12.1$ eV^36^ with asymptotic 6 photon absorption coefficient of $1.6\times 10^{-71}\,\hbox {cm}^9$/$\hbox {W}^5$. Free electron generation was simulated using a rate equation describing the evolution of the density of electrons in the conduction band:

4 $$\begin{aligned} \frac{\partial \rho }{\partial t}= W\left( 1-\frac{\rho }{\rho _{nt}}\right) + \frac{\sigma }{U_g}|E|^2\rho \left( 1-\frac{\rho }{\rho _{nt}}\right) -\frac{\rho }{\tau _{rec}}, \end{aligned}$$

where the terms on the right hand side stand for photoionization, avalanche ionization and recombination, respectively, where $\tau _{rec}=150$ fs is the free electron recombination time.

## Results and discussion

**Figure 2:**
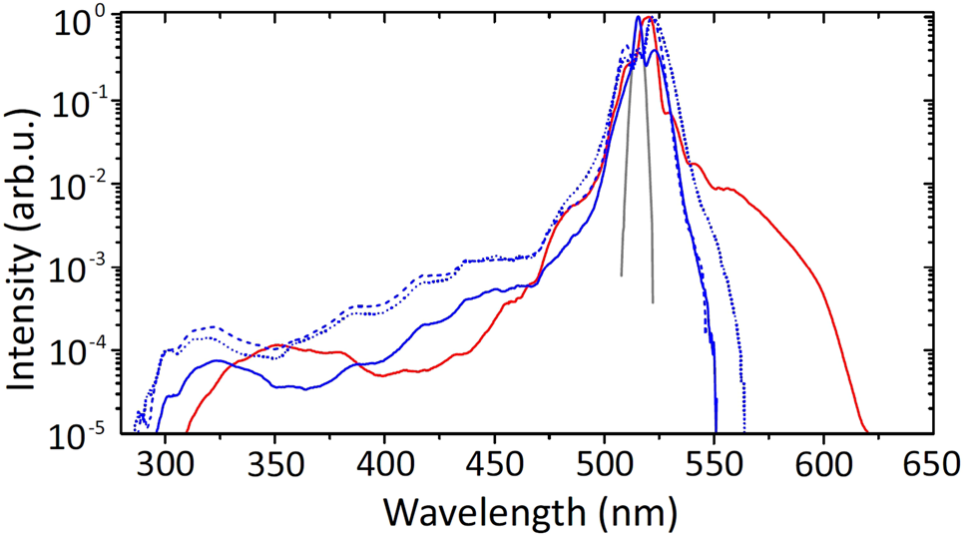
Supercontinuum spectra generated in $\hbox {CaF}_{{2}}$ samples of 5 mm with $\hbox {NA}=0.012$ (blue curve) and 25 mm with $\hbox {NA}=0.004$ (red curve). The dotted and dashed blue curves show the supercontinuum spectra generated in 25 mm long $\hbox {CaF}_{{2}}$ sample with $\hbox {NA}=0.012$, using focusing configurations depicted in Fig. 1c,d, respectively. The pump pulse spectrum is shown by a grey curve.

Figure 2 shows single-shot SC spectra measured using the focusing configurations depicted in Fig. 1. The measurements in tight and loose focusing geometries were performed using pump pulse energies of $0.40\,\mu \hbox {J}$ and $0.48\,\mu \hbox {J}$, respectively. In all cases the SC spectra exhibit very similar blue-shifted spectral broadenings with the shortwave cut-off wavelengths (defined at the $10^{-5}$ intensity level) of 296 nm (tight focusing, thin sample), 290 nm (tight focusing, thick sample, both configurations) and 309 nm (loose focusing, thick sample), yielding the respective blue shifts of $14370 \,\hbox {cm}^{-1}$, $15065\, \hbox {cm}^{-1}$ and $12940 \,\hbox {cm}^{-1}$. However, configurations that employ tight focusing into either thin or thick sample, produce considerably smaller broadening on the long wavelength side compared to loose focusing case into a thick sample, which produces well-pronounced red-shifted spectral broadening up to 620 nm, consistent with the earlier observations of enhanced spectral red-shifts reported in loose focusing conditions, in e.g., sapphire^29,30^.

**Figure 3:**
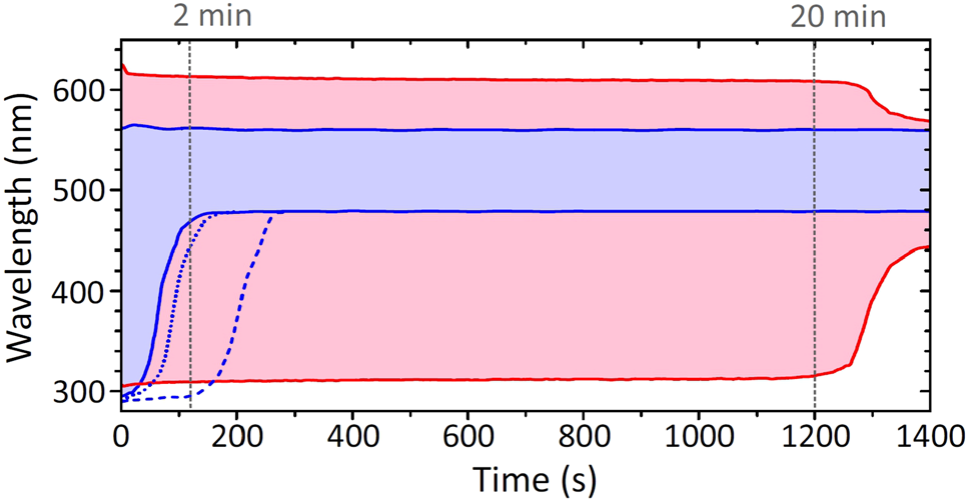
The time evolutions of the SC spectral width in $\hbox {CaF}_{{2}}$. Blue area: tight focusing ($\hbox {NA}=0.012$) into a short (5 mm) sample, red area: loose focusing ($\hbox {NA}=0.004$) into a long (25 mm) sample. The dotted and dashed contours show the time evolutions of the SC spectral widths measured by tight focusing into 25 mm long $\hbox {CaF}_{{2}}$ sample, using focusing configurations depicted in Fig. 1c,d, respectively.

**Figure 4:**
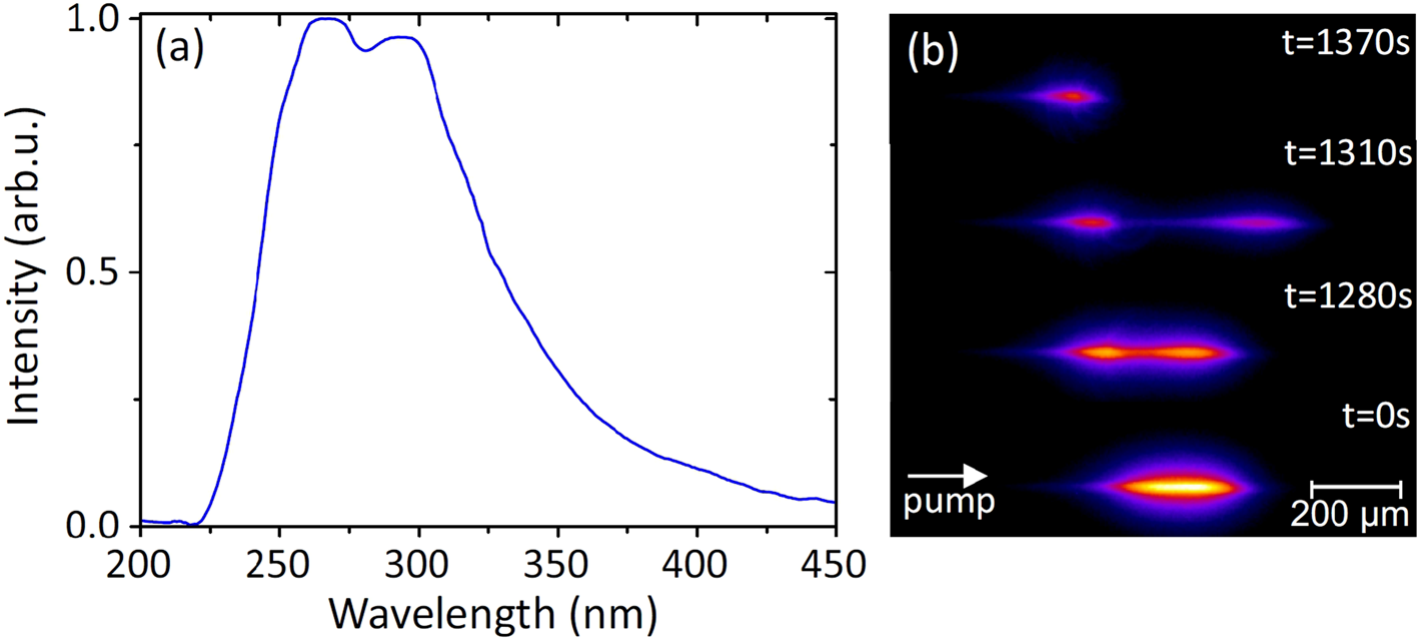
(**a**) Filament-induced luminescence spectrum in $\hbox {CaF}_{{2}}$. (**b**) Examples of filament-induced luminescence traces recorded at different exposure times. The propagation direction of the beam is indicated by an arrow.

Tight and loose focusing geometries produced remarkably different evolutions of the SC spectral width in time. Figure 3 compares the time evolutions of SC spectral bandwidths (defined by the boundary shortwave and longwave cut-off wavelengths at the $10^{-5}$ intensity level) in focusing configurations shown in Fig. 1. In performing this measurement, the operating conditions were identical to those described above and the samples were kept at a stationary position, i.e. not translated with respect to the pump beam. In the case of tight focusing into a thin sample, the shrinking of SC spectrum becomes apparent almost immediately (in a few seconds), and SC spectrum completely extinguishes after 2 min of continuous exposure at a 10 kHz laser repetition rate. Tight focusing into 25 mm long sample shows very similar result. In contrast, much more durable SC generation performance was observed in the case of loose focusing into a thick sample: despite a very slight gradual shrinking (by 5 nm on the short wavelength side and 15 nm on the long wavelength side), fairly stable SC generation is observed for 20 min (1200 s), until considerable shrinking of its spectrum starts to be clearly detectable. It is worth mentioning that much worse (in terms of durability) performance was observed using the pump pulses at fundamental harmonic wavelength (1035 nm). Even using loose focusing into a long sample, the measured SC blue cut-off wavelength was at 350 nm (at the $10^{-4}$ intensity level) and extinction of the SC spectrum was observed after just $\sim 3$ min of exposure time, affirming our motivation to use the pump pulses at the second harmonic wavelength instead of fundamental.

In what follows, we examine the case of loose focusing into a thick sample in more detail by performing simultaneous measurements of filament-induced luminescence trace, which provides the information on the shape of the light filament, and light scattering at the pump wavelength, which indicates the onset of the optical damage. The filament-induced luminescence is attributed to self-trapped exciton emission in the UV^37^, which exhibits a broad spectrum with a peak intensity in the $\sim 260-300$ nm range, shown in Fig. 4a. Figure 4b presents illustrative examples of the filament-induced luminescence traces, recorded at different exposure times. The luminescence trace at the bottom of the figure ($t=0$ denotes the beginning of measurement) attests formation of a single filament, whose the most intense part indicates the position of nonlinear focus which is located $\sim 8$ mm from the exit face of the sample. The above luminescence traces were recorded at exposure times, where extinction of SC spectrum occurs. These reveal break-up of the filament at $\hbox {t} = 1280$ s, thereafter the increased separation between its detached parts at $\hbox {t} = 1310$ s and eventually, the decay of the filament at $\hbox {t} = 1370$ s, where only a short and faint fragment of luminescence at its leading front remains detectable (note the propagation direction indicated by an arrow), suggesting a correlation between the extinction of SC spectrum and break-up and decay of the light filament.

**Figure 5:**
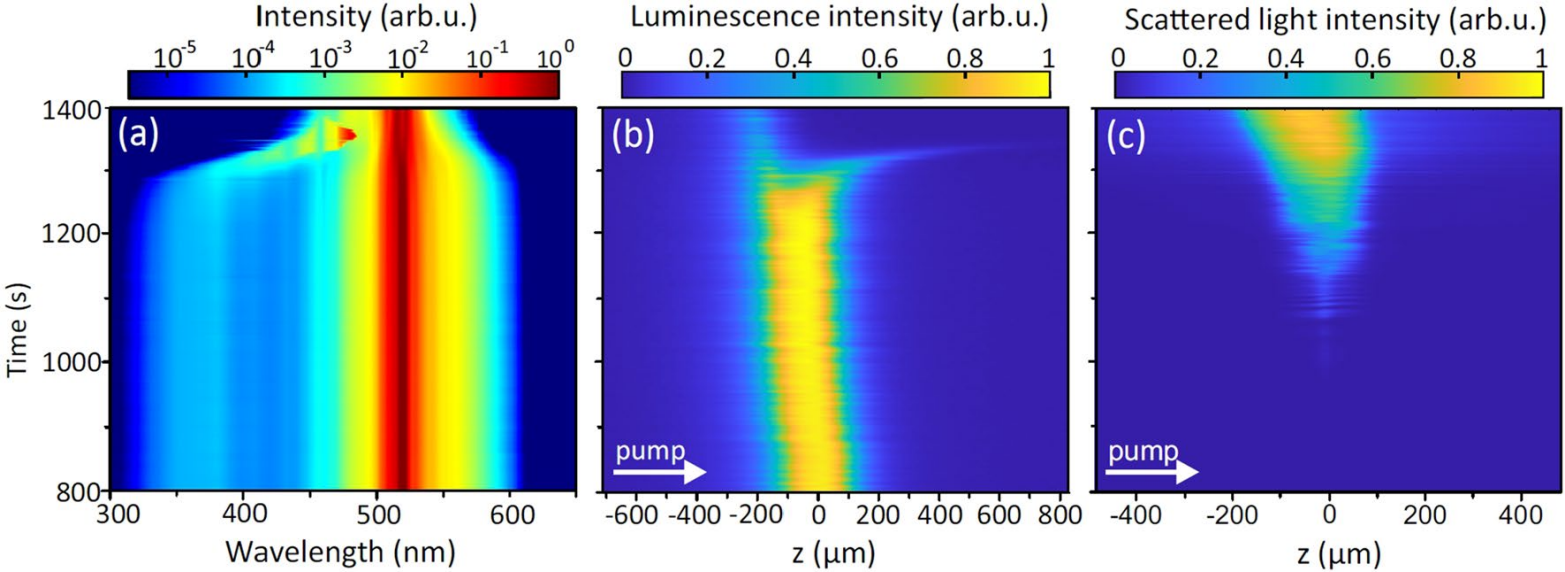
The time evolutions of (**a**) supercontinuum spectrum, (**b**) filament-induced luminescence trace and (**c**) intensity of scattered light in a thick (25 mm) $\hbox {CaF}_{{2}}$ slab measured with loosely focused ($\hbox {NA} = 0.004$) 150 fs, 515 nm pulses at a 10 kHz repetition rate. *z* denotes the longitudinal coordinate.

Figure 5 presents more detailed time evolutions of SC spectrum, UV luminescence trace and scattering signal at the pump wavelength (515 nm) within the time interval where relevant changes in SC spectrum occur. *z* is the longitudinal coordinate in Fig. 5b,c, where $\hbox {z} = 0$ corresponds to the center of luminescence trace at the beginning of measurement. Interestingly, the signal of scattered light is detected slightly before ($\sim 200$ s) any noticeable changes of the SC spectrum and the shape of filament-induced luminescence trace occur. In fact, the occurrence of light scattering indicates the onset of the optical damage at its early stage, which first occurs as defect accumulation leading to a structural modification of the material thereafter, see e.g.^38^, at the site of nonlinear focus (where the beam intensity due to self-focusing is the highest), and the scattered light intensifies with damage becoming catastrophic. More specifically, the electronic excitation energy acquired by the multiphoton absorption (more precisely, six photon absorption, assuming the incident photon energy of 2.4 eV and material bandgap of 12.1 eV) and inverse Bremsstrahlung effect is localized by fast (on the sub-picosecond time scale) creation of self-trapped excitons^39^, whose lifetime is considerably longer^40^. The radiative decay of self-trapped excitons is observed as filament-induced luminescence, see Fig. 4, while non-radiative decay of self-trapped excitons results in production of long lived color centers, which serve as point defects (lattice distortions)^41^. These point defects act as scattering centers on the microscopic scale, while in macroscopic scale no apparent changes in the filament-induced luminescence trace and SC spectrum are yet detectable. The optical damage is initiated through formation of a nanograting^42^, resulting from accumulation of point defects with every following laser pulse. In that way, the modified volume along the filament path expands, resulting in break-up of the light filament. Eventually, nanograting transforms into catastrophic optical damage, which causes severe absorption and light scattering, so terminating formation of the light filament and subsequently, the SC generation. Very similar time evolutions of SC spectrum, UV luminescence trace and scattering signal were observed in the case of tight focusing into a short sample, however in this case the relevant changes occur on much shorter time scale, and filament formation is completely terminated after 2 min of exposure time.

**Figure 6:**
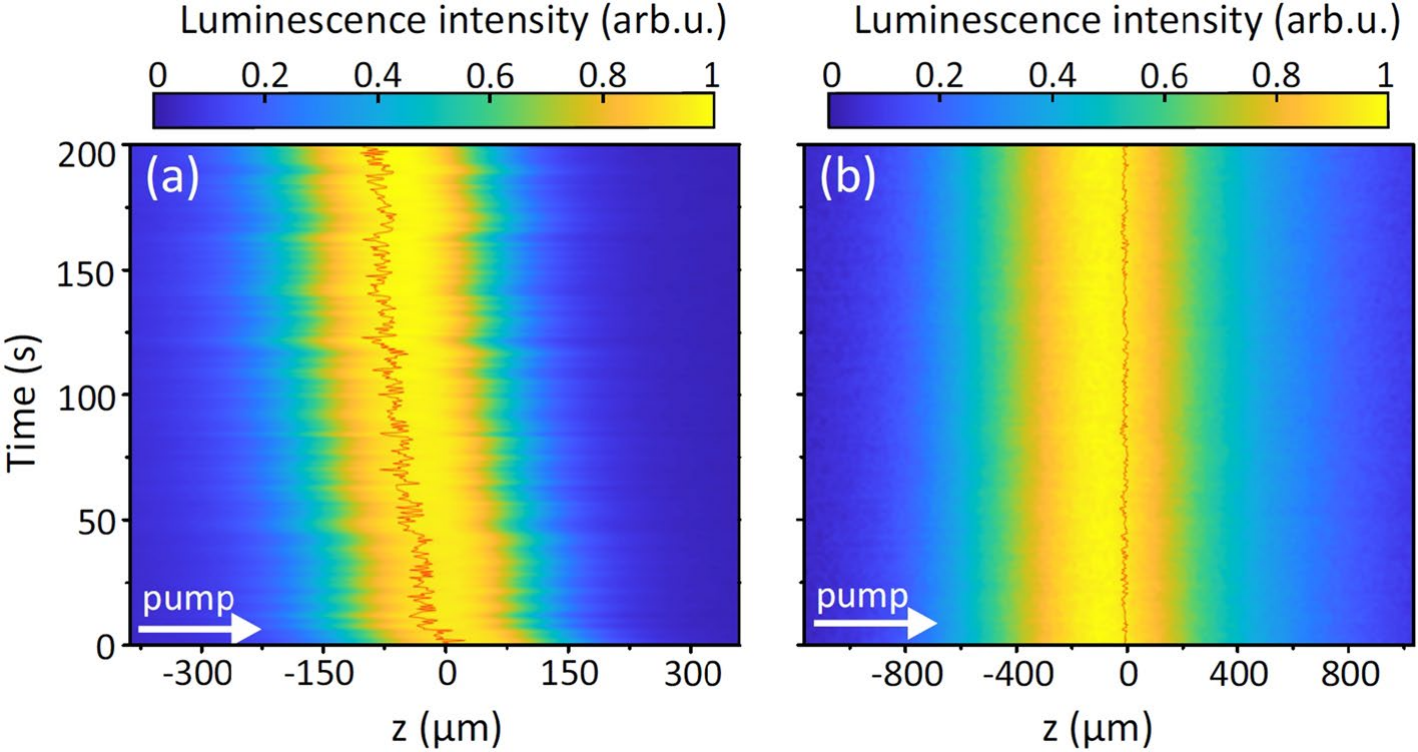
The time evolutions of the filament-induced luminescence traces of loosely ($\hbox {NA} = 0.004$) focused pump pulses in: (**a**) $\hbox {CaF}_{{2}}$ sample of 25 mm thickness (the pump pulse energy of $0.48\,\mu \hbox {J}$), (**b**) sapphire sample of 15 mm thickness (the pump pulse energy of $0.34\,\mu \hbox {J}$) at a 10 kHz repetition rate. Red curves indicate the calculated centers of mass of luminescence traces.

A closer inspection of the time evolution of filament-induced luminescence trace shows its slight but gradual drift toward the entrance face of the sample (toward negative *z* values) even in the case of apparently stable SC generation performance, where no light scattering is detected and no spectral narrowing of SC is observed. Moreover, the filament-induced luminescence trace exhibits continuous longitudinal jittering, as evident from Fig. 6a, which presents the dynamics of the filament-induced luminescence trace from the beginning of measurement. We verified experimentally that these features are not related to the energy drift or pulse-to-pulse instability of the driving laser (the measured standard deviation of laser pulse energy was 0.3%, without any detectable long-term energy drift). Therefore we performed a complimentary experiment by replacing $\hbox {CaF}_{{2}}$ crystal with undoped sapphire crystal of 15 mm thickness, while keeping identical loose focusing condition ($\hbox {NA}=0.004$) of the pump beam. The light filament in sapphire induces a broadband luminescence with a center wavelength of 280 nm^43^, which is attributed to F center emission^44^. Figure 6b presents the time evolution of filament-induced luminescence trace in sapphire, with the pump pulse energy of $0.34~\mu$J, which produced a stable SC spectrum spanning from 390 to 700 nm (at the $10^{-5}$ intensity level). In contrast to $\hbox {CaF}_{{2}}$, the filament-induced luminescence trace in sapphire keeps its position fixed and shows no jittering, as attested also by a constant position of calculated center of mass of the luminescence trace at any moment of time. However, although sapphire shows extremely stable, damage-free performance on a long term, it produces only very modest spectral broadening into the UV.

The origin of jittering of the luminescence trace in $\hbox {CaF}_{{2}}$ could be attributed to a dynamic interplay between the rates of color center formation via decay of self-trapped excitons and color center extinction due to local heating of the material. In contrast, the lifetime of F centers in sapphire is assumed to be very short and they decay well before the next laser pulse arrives. The observed continuous drift of the filament position toward the entrance face of $\hbox {CaF}_{{2}}$ sample could be explained by the following considerations. Since the energy band of color centers is located at 3.0 eV^39^, they absorb the pulse energy with a higher probability due to lower order of multiphoton absorption (two photon absorption) than pristine material does, hence slightly affecting the nonlinear propagation of every subsequent laser pulse. In that way, the next laser pulse deposits slightly larger amount of energy to the material (experiences slightly larger net nonlinear absorption), resulting in production of a slightly larger number of free electrons, which in turn stimulate further production of color centers. So with every next arriving pulse, the forefront of the plasma channel and so the forefront of the luminescence track crawls toward the entrance face of the material. The same considerations apply also to the position of the scattering site, which apparently crawls toward the entrance face of the material as well, see Fig. 5c.

**Figure 7:**
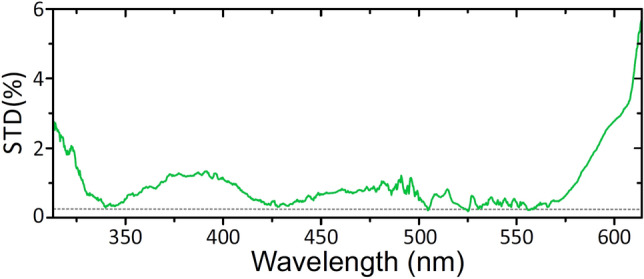
Standard deviation of spectral intensity fluctuations across the supercontinuum spectrum generated in 25 mm thick $\hbox {CaF}_{{2}}$ crystal at loose focusing condition ($\hbox {NA}=0.004$). The dotted line marks standard deviation of the pump pulse energy.

Despite continuous jittering of the filament position in $\hbox {CaF}_{{2}}$, a fairly stable SC radiation at the crystal output is produced. Figure 7 shows the standard deviation of spectral intensity fluctuations across the entire SC spectrum. The largest intensity fluctuations (exceeding 2%) were measured for the most blue-shifted (for wavelengths $<320$ nm) and red-shifted (for wavelengths $>600$ nm) spectral components, where SC intensity rapidly drops. The spectral intensity fluctuations within the major and the most intense part of the SC spectrum were generally below 1%, with the standard deviation of the pump pulse energy of 0.3% provided by the laser source. The certain zones of very high stability around the wavelengths of 340 nm, 430 nm and around the pump wavelength (515 nm) nearly matching the stability of the laser source could be attributed to complex nature of spectral and temporal correlations between spectral components of the SC radiation^45,46^.

**Figure 8:**
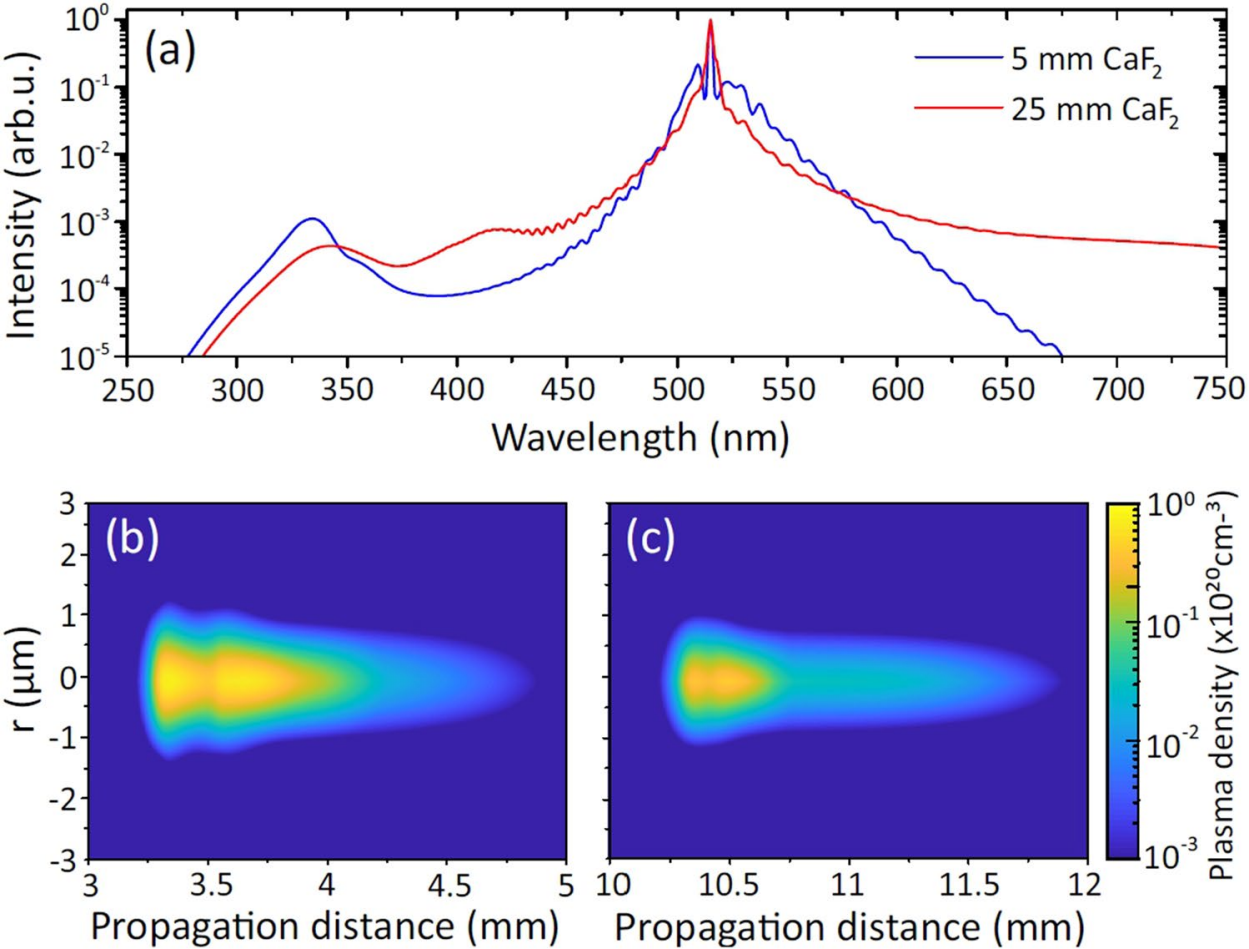
(**a**) Numerically simulated SC spectra in tight and loose focusing conditions and calculated plasma densities around the nonlinear focus, corresponding to (**b**) tight and (**c**) loose focusing conditions of the pump beam. *r* denotes the radial coordinate.

Finally, in order to explain the large difference in durability of the nonlinear material in the cases of tight and loose focusing, we performed the numerical simulations of nonlinear propagation and filament formation under conditions that correspond to the experimental settings (pulsewidth, energy) and focusing configurations depicted in Fig. 1a,b. Figure 8a shows the numerically simulated SC spectra in tight and loose focusing conditions, after the nonlinear propagation in $\hbox {CaF}_2$ samples of 5 mm and 25 mm thickness, respectively. Although the numerical simulation yields generally broader SC spectra, they nevertheless qualitatively share the relevant features (a slight difference in shortwave cut-offs and a remarkable difference in spectral red-shifts) with experimentally measured SC spectra shown in Fig. 2.

Although the numerical simulations were performed in the case of a single pulse propagation and do not account for any accumulative effects, the results nevertheless give useful insight into the differences of plasma production in tight and loose focusing conditions. Figure 8b,c compare the maximum plasma densities produced by a light filament in the cases of interest. Tight focusing of the pump beam yields a maximum plasma density of $7.3\times 10^{19}\,\hbox {cm}^{-3}$, which is $\sim 1.7$ times larger than that is produced in loose focusing condition ($4.2\times 10^{19}\,\hbox {cm}^{-3}$). We consider this difference relevant, since formation of long-lived color centers strongly depends on local plasma density, especially concerning the accumulative character of color center formation by repetitive laser pulses and the speed at which the subsequent optical damage evolves.

## Conclusions

In conclusion, we studied supercontinuum generation in calcium fluoride ($\hbox {CaF}_2$) with green (515 nm) femtosecond (150 fs) pulses at a repetition rate of 10 kHz. We demonstrate that loose focusing ($\hbox {NA}=0.004$) of the pump beam into a long (25 mm) $\hbox {CaF}_2$ sample produces a nearly symmetrically broadened supercontinuum spectrum which remains stable in untranslated crystal for 20 minutes of operation. We show that this supercontinuum generation geometry has an advantage over setups which apply relatively tight focusing ($\hbox {NA}=0.012$), where mostly blue-shifted supercontinuum spectrum is produced and extinguishes in a few minutes due to optical degradation of the crystal, regardless of its length and focusing condition. The large difference in durability of damage-free operation of the nonlinear material in tight and loose focusing conditions is explained by the results of numerical simulations, suggesting that tight focusing into a short sample initially produces almost twice larger free electron density at the nonlinear focus of the beam, thereby promoting formation and faster accumulation of long-lived color centers. The simultaneous measurements of time evolutions of supercontinuum spectrum, filament-induced luminescence due to self-trapped exciton emission and light scattering indicating the onset of optical damage demonstrate how the propagation of light filament and its spectral broadening are altered by evolving optical damage of the nonlinear material. In particular, it is shown that even in the case of apparently stable performance with loose focusing, filament-induced luminescence trace exhibits continuous longitudinal jittering, which is attributed to the dynamical interplay between formation and extinction of color centers due to nonlinear propagation of intense laser pulse.

Our results could be of practical importance for optimizing the SC generation setups which aim at production of SC spectrum with considerable UV extent in alkali metal fluoride crystals, those, as a rule, suffer from rapid optical degradation due to formation of long-lived color centers that lead to catastrophic optical damage of these materials.

## References

[1] Nagura C, Suda A, Kawano H, Obara M, Midorikawa K (2002). Generation and characterization of ultrafast white-light continuum in condensed media. Appl. Opt..

[2] Dubietis A, Tamošauskas G, Šuminas R, Jukna V, Couairon A (2017). Ultrafast supercontinuum generation in bulk condensed media. Lith. J. Phys..

[3] Dubietis A, Couairon A (2019). Ultrafast Supercontinuum Generation in Transparent Solid State Media.

[4] Brodeur A, Chin SL (1998). Band-gap dependence of the ultrafast white-light continuum. Phys. Rev. Lett..

[5] Kolesik M, Katona G, Moloney JV, Wright EM (2003). Physical factors limiting the spectral extent and band gap dependence of supercontinuum generation. Phys. Rev. Lett..

[6] Tzankov P, Buchvarov I, Fiebig T (2002). Broadband optical parametric amplification in the near UV-VIS. Opt. Commun..

[7] Dharmadhikari AK, Rajgara FA, Reddy NCS, Sandhu AS, Mathur D (2004). Highly efficient white light generation from barium fluoride. Opt. Express.

[8] Johnson PJM, Prokhorenko VI, Miller RJD (2009). Stable UV to IR supercontinuum generation in calcium fluoride with conserved circular polarization states. Opt. Express.

[9] Kohl-Landgraf J, Nimsch J-E, Wachtveitl J (2013). LiF, an underestimated supercontinuum source in femtosecond transient absorption spectroscopy. Opt. Express.

[10] Darginavičius J (2013). Ultrabroadband supercontinuum and third-harmonic generation in bulk solids with two optical-cycle carrier-envelope phase-stable pulses at $$2~\mu$$m. Opt. Express.

[11] Dharmadhikari JA (2014). Effect of group velocity dispersion on supercontinuum generation and filamentation in transparent solids. Appl. Phys. B.

[12] Dormidonov AE, Kompanets VO, Chekalin SV, Kandidov VP (2015). Giantically blue-shifted visible light in femtosecond mid-IR filament in fluorides. Opt. Express.

[13] Liang H (2015). Three-octave-spanning supercontinuum generation and sub-two-cycle self-compression of mid-infrared filaments in dielectrics. Opt. Lett..

[14] Garejev N (2016). Odd harmonics-enhanced supercontinuum in bulk solid-state dielectric medium. Opt. Express.

[15] Garejev N, Tamošauskas G, Dubietis A (2017). Comparative study of multioctave supercontinuum generation in fused silica, YAG, and LiF in the range of anomalous group velocity dispersion. J. Opt. Soc. Am. B.

[16] Wang J, Zhang Y, Shen H, Jiang Y, Wang Z (2017). Spectral stability of supercontinuum generation in condensed mediums. Opt. Eng..

[17] Marcinkevičiūtė A, Garejev N, Šuminas R, Tamošauskas G, Dubietis A (2017). A compact, self-compression-based sub-3 optical cycle source in the $$3-4~\mu$$m spectral range. J. Opt..

[18] Chekalin SV, Dormidonov AE, Kompanets VO, Zaloznaya ED, Kandidov VP (2019). Light bullet supercontinuum. J. Opt. Soc. Am. B.

[19] Kryukov IV, Petrov Nkh, Alfimov MV (2020). A supercontinuum generator with pumping by pulses of chromium-forsterite-based femtosecond laser in transparent condensed media. Instrum. Exp. Tech..

[20] Šuminiene A (2020). LiSAF: an efficient and durable nonlinear material for supercontinuum generation in the ultraviolet. Lith. J. Phys..

[21] Zaloznaya E (2021). Short wavelength cutoff of the light bullet spectrum in calcium fluoride. Appl. Phys. B.

[22] Gallais L, Commandré M (2014). Laser-induced damage thresholds of bulk and coating optical materials at 1030 nm, 500 fs. Appl. Opt..

[23] Marcinkevičiūtė A (2019). Supercontinuum generation in the absence and in the presence of color centers in NaCl and KBr. Res. Phys..

[24] Ziolek M, Naskrecki R, Karolczak J (2004). Some temporal and spectral properties of femtosecond supercontinuum important in pump-probe spectroscopy. Opt. Commun..

[25] Megerle U, Pugliesi I, Schriever C, Sailer CF, Riedle E (2009). Sub-50 fs broadband absorption spectroscopy with tunable excitation: Putting the analysis of ultrafast molecular dynamics on solid ground. Appl. Phys. B.

[26] Krebs N, Pugliesi I, Hauer J, Riedle E (2013). Two-dimensional Fourier transform spectroscopy in the ultraviolet with sub-20 fs pump pulses and 250–720 nm supercontinuum probe. New. J. Phys..

[27] Huber R, Satzger H, Zinth W, Wachtveitl J (2001). Noncollinear optical parametric amplifiers with output parameters improved by the applications of a white light continuum generated in $$\text{ CaF}_2$$. Opt. Commun..

[28] Tzankov P, Fiebig T, Buchvarov I (2003). Tunable femtosecond pulses in the near-ultraviolet from ultrabroadband parametric amplification. Appl. Phys. Lett..

[29] Bradler M, Baum P, Riedle E (2009). Femtosecond continuum generation in bulk laser host materials with sub-$$\mu$$J pump pulses. Appl. Phys. B.

[30] Jukna V, Galinis J, Tamošauskas G, Majus D, Dubietis A (2014). Infrared extension of femtosecond supercontinuum generated by filamentation in solid-state media. Appl. Phys. B.

[31] Indra L (2017). Picosecond pulse generated supercontinuum as a stable seed for OPCPA. Opt. Lett..

[32] Riedle E, Bradler M, Wenninger M, Sailer CF, Pugliesi I (2013). Electronic transient spectroscopy from the deep UV to the NIR: Unambiguous disentanglement of complex processes. Faraday Discuss..

[33] Couairon A (2011). Practitioners guide to laser pulse propagation models and simulation. Eur. Phys. J. Special Top..

[34] Malitson IH (1963). A redetermination of some optical properties of calcium fluoride. Appl. Opt..

[35] Kabaciński P, Kardaś TM, Stepanenko Y, Radzewicz C (2019). Nonlinear refractive index measurement by SPM-induced phase regression. Opt. Express.

[36] Rubloff GW (1972). Far-ultraviolet reflectance spectra and the electronic structure of ionic crystals. Phys. Rev. B.

[37] Cramer LP, Cumby TD, Leraas JA, Langford SC, Dickinson JT (2005). Effect of surface treatments on self-trapped exciton luminescence in single-crystal $$\text{ CaF}_2$$. J. Appl. Phys..

[38] Richter S, Heinrich M, Döring S, Tünnermann A, Nolte S (2011). Formation of femtosecond laser-induced nanogratings at high repetition rates. Appl. Phys. A.

[39] Lindner R, Reichling M, Williams RT, Matthias E (2001). Femtosecond laser pulse excitation of electrons and excitons in $$\text{ CaF}_2$$ and $$\text{ SrF}_2$$. J. Phys. Condens. Matter.

[40] Stellmer S, Schreitl M, Schumm T (2015). Radioluminescence and photoluminescence of Th:$$\text{ CaF}_2$$ crystals. Sci. Rep..

[41] Mao SS (2004). Dynamics of femtosecond laser interactions with dielectrics. Appl. Phys. A.

[42] Richter S (2012). Nanogratings in fused silica: Formation, control, and applications. J. Laser Appl..

[43] Grigutis R, Tamošauskas G, Jukna V, Risos A, Dubietis A (2020). Supercontinuum generation and optical damage of sapphire and YAG at high repetition rates. Opt. Lett..

[44] Caulfield KJ, Cooper R, Boas JF (1993). Luminescence from electron-irradiated sapphire. Phys. Rev. B.

[45] Majus D, Dubietis A (2013). Statistical properties of ultrafast supercontinuum generated by femtosecond Gaussian and Bessel beams: A comparative study. J. Opt. Soc. Am. B.

[46] Bradler M, Riedle E (2014). Temporal and spectral correlations in bulk continua and improved use in transient spectroscopy. J. Opt. Soc. Am. B.

